# Analysis of signs and symptoms in confirmed cases of severe dengue among children aged 0 to 10 years old

**DOI:** 10.31744/einstein_journal/2024AO0546

**Published:** 2024-04-10

**Authors:** Álvaro Silvério Avelino da Silva, Francisco Leal Carvalho, Giovanna Araújo Pinto, Laís Silva Rios Saad, Mariana Oliveira Curado, Thais Caroline Dallabona Dombroski, Hugo Dias Hoffmann-Santos, Rosa Maria Elias

**Affiliations:** 1 Hospital São Vicente de Paulo Brasília DF Brazil Hospital São Vicente de Paulo, Brasília, DF, Brazil.; 2 Centro Universitário de Várzea Grande Várzea Grande MT Brazil Centro Universitário de Várzea Grande, Várzea Grande, MT, Brazil.

**Keywords:** Dengue, Child, Epidemiology, Signs and symptoms, Brazil

## Abstract

**Objective:**

The prevalent symptoms of severe dengue in pediatric patients are divided into three subgroups: severe plasma leakage, severe bleeding, and severe organ damage. In addition, the seasonal patterns of the disease and the outcomes of cure or death from dengue were evaluated.

**Methods:**

An epidemiological, observational, analytical, cross-sectional study was conducted with data from the Notifiable Disease Information System (SINAN - *Sistema de Informação de Agravos de Notificação* and DATASUS - *Departamento de Informática do Sistema Único de Saúde*) of the Ministry of Health from 2019 to 2020.

**Results:**

During the study period, 1,857 cases of severe dengue were observed in the pediatric age group, with the most common symptoms being respiratory failure, melena, hematemesis, and altered level of consciousness. The total proportion of patients hospitalized for severe dengue was 89.6%, and 51.2% of these patients died, corroborating the importance of early detection of the disease.

**Conclusion:**

Severe dengue is more prevalent during the seasonal period, with hot and humid characteristics owing to the mechanism involved in the viral cycle. The most prevalent symptoms of severe dengue in pediatric patients were respiratory failure alone, gastrointestinal bleeding, and altered level of consciousness. It is important to identify signs of severity for early intervention and a better prognosis, considering that death is closely related to a delayed diagnosis.

## In Brief

This was an epidemiological, analytical, and cross-sectional study using data from SINAN from 2019 to 2020 that analyzed the symptoms of severe dengue, its seasonal patterns, and the progression to cure or death. We found a total of 1,857 patients with respiratory failure, gastrointestinal bleeding, altered level of consciousness, and hospitalization. The disease is most common in the hot and humid periods and early detection is key to prevent death.

## Highlights

▪ The average age was 4.34±2.56 years.
▪ Highest prevalence in Brown and White races.
▪ Highest prevalence rate was found in the Southeast, followed by the Central-West and South.
▪ The majority of analyzed cases progressed to hospitalization.
▪ Prevalent respiratory failure, hematemesis, and melena are associated with both.
▪ Prevalence of changes in the level of consciousness, which is important at the central nervous system level.

## Graphical abstract

**Figure:**
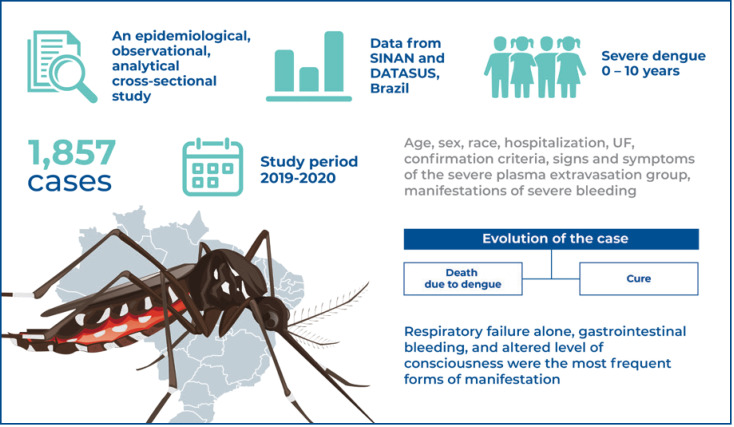

## INTRODUCTION

Dengue, an arbovirus transmitted by the *Aedes aegypti* mosquito, is considered a serious public health problem in Brazil.^(1)^ It was initially described in the 1990s as a disease with a seasonal pattern, with a higher incidence in the hot and humid months, mainly from January to May.^(2)^

In 2015 and 2016, a dengue epidemic occurred in Brazil^(2)^ which can be explained by the failure to control the vector mosquito population, the increase in population and urbanization, low incomes, excessive garbage, climate change, and the growth in the number of international travelers, among others.^(2,3)^ The Midwest region had the highest incidence rate (536.4 cases/100,000 inhabitants), followed by the South (218.1/100,000), and Southeast (209.9/100,000).^(4)^ It is notable that in the last decade, at least 25% of individuals reported and hospitalized for Dengue were under 15 years old.^(5)^

Dengue transmission occurs through the bite of a mosquito infected with the known serotypes: DENV 1, 2, 3, or 4^(1,6)^ and can manifest in the classic form (with or without warning signs) or the severe form. The classification of severe dengue includes one or more of the following groups of symptoms: plasma leakage (signs of shock and fluid accumulation), bleeding (melena, hematemesis, metrorrhagia and central nervous system (CNS) bleeding) or severe organ impairment (altered level of consciousness, myocarditis, altered liver enzymes).^(7)^

The severe form is most worrisome because the leakage of fluids and intravascular coagulation disorders can lead to organ dysfunction due to tissue hypoperfusion.^(8)^ According to the Brazilian Society of Pediatrics (SBP - *Sociedade Brasileira de Pediatria*), infants are more likely to present with serious disease, as are White people, and those with chronic diseases.^(7)^

Children have hematological alterations that are peculiar to cases of severe dengue, which also occur in other groups, such as pregnant women, patients with hemophilia, those hospitalized in intensive care units, and patients infected with two serotypes at the same time. The pathophysiology is related to the degree of viremia, as it triggers the activation of the cytokine cascade and complement complex. This accentuated stimulus causes endothelial dysfunction, coagulation factor consumption, and platelet destruction with consequent plasma leakage and hemorrhagic manifestations. Complications occur due to endothelial dysfunction, which causes defervescence, hemodynamic changes, serous effusion, and organ dysfunction.^(9)^

## OBJECTIVE

To analyze the most prevalent pediatric symptoms of severe dengue which was divided into three subgroups: severe plasma leakage, severe bleeding, and severe organ damage. In addition, the seasonal patterns of the disease and the outcomes of cure or death from dengue were evaluated.

## METHODS

An epidemiological, observational, analytical cross-sectional study was conducted with data from the Notifiable Disease Information System (SINAN - *Sistema de Informação de Agravos de Notificação and* DATASUS - *Departamento de Informática do Sistema Único de Saúde*) regarding confirmed cases of severe dengue that occurred in all units of the Brazilian federation, from 2019 to 2020, among individuals aged 0-10 years.

The following variables were included: year of notification, federal unit of notification, age, sex, race, hospitalization, infection status, classification, confirmation criteria, case evolution (death due to dengue or cure), weak or undetectable pulse, convergent blood pressure (BP), capillary refill time, fluid accumulation with respiratory failure, tachycardia, cold extremities, hypotension in the late phase, hematemesis, melena, massive metrorrhagia, central nervous system bleeding, aspartate aminotransferase (AST)/alanine aminotransferase (ALT) >1000, myocarditis and alteration of consciousness.

Categorical variables are summarized using absolute (n) and relative (%) frequencies, and continuous variables are summarized using means and standard deviations. The association between categorical variables was assessed using Pearson’s chi-square test, and the odds ratio was considered a measure of association, followed by a 95% confidence interval. Statistical significance was set at p<0.05, and all analyses were performed using Jamovi software version 2.3 (https://www.jamovi.org).

## RESULTS

During the study period, 1,857 cases of severe dengue were reported in the pediatric age group, with a mean age of 4.34±2.56 years. There was no significant distinction regarding the sex of patients (Table 1).

**Table 1:** Data on pediatric patients diagnosed with severe dengue

Variables		n (%)
Sex		
	Male	908 (48.9)
	Female	949 (51.1)
Race		
	Brown	779 (41.9)
	White	950 (51.3)
	Black	90 (4.8)
	Yellow	28 (1.5)
	Indigenous	10 (0.5)
Age		
	0	155 (8.3)
	1	176 (9.5)
	2	196 (10.6)
	3	193 (10.4)
	4	213 (11.5)
	5	210 (11.3)
	6	257 (13.8)
	7	243 (13.1)
	8	167 (9)
	9	44 (2.4)
	10	3 (0.2)
Hospitalization		
	Yes	1,589 (89.6)
	No	185 (10.4)
	Missing	83
Federal Unit Infections		
	Acre	9 (0.6)
	Alagoas	32 (2)
	Amazonas	13 (0.8)
	Bahia	40 (2.6)
	Ceará	22 (1.4)
	Distrito Federal	53 (3.4)
	Espírito Santo	80 (5.1)
	Goiás	159 (10.2)
	Maranhão	37 (2.4)
	Mato Grosso	49 (3.1)
	Mato Grosso Do Sul	65 (4.2)
	Minas Gerais	164 (10.5)
	Pará	1 (0.1)
	Paraíba	9 (0.6)
	Paraná	281 (18)
	Pernambuco	9 (0.6)
	Piauí	19 (1.2)
	Rio de Janeiro	4 (0.3)
	Rio Grande do Norte	11 (0.7)
	Rio Grande do Sul	3 (0.2)
	Rondônia	1 (0.1)
	Roraima	7 (0.4)
	Santa Catarina	3 (0.2)
	São Paulo	452 (28.9)
	Sergipe	26 (1.7)
	Tocantins	13 (0.8)
	Missing	295

There was a higher prevalence among the Brown and White patients when compared to the other groups. Regarding the demographic distribution, the Southeast region had the highest rate of severe dengue cases during the study period, followed by the Midwest and South regions. Laboratory tests were the main criterion for the diagnostic confirmation of dengue. Among patients with severe dengue, 89.6% were hospitalized.

**Figure 1:**
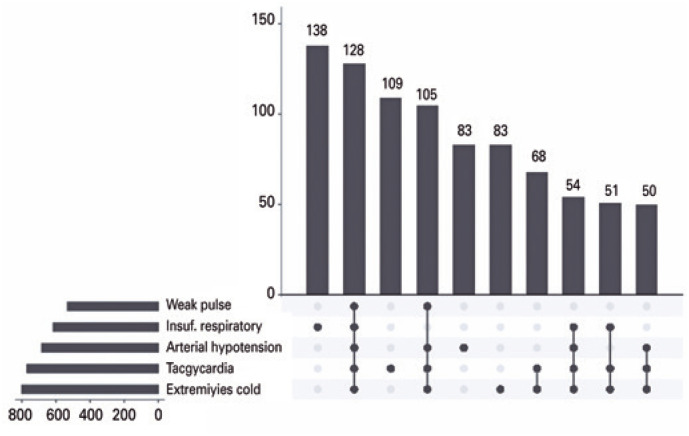
Combination analysis of signs and symptoms of severe plasma leakage in pediatric patients with severe dengue in Brazil

**Figure 2:**
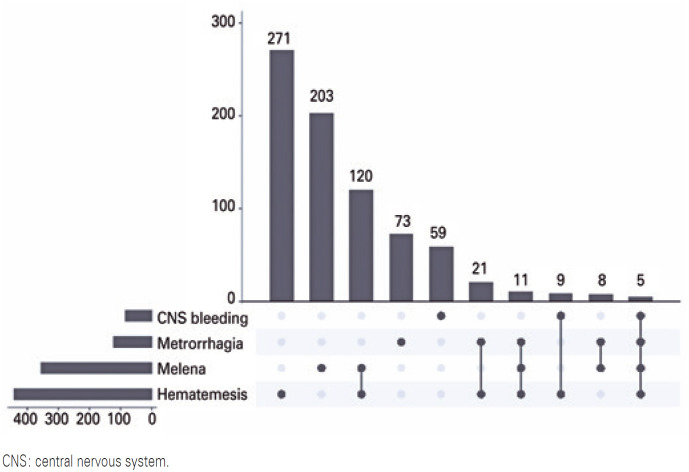
Combination analysis of signs and symptoms of severe plasma bleeding in pediatric patients with severe dengue in Brazil

The SINAN Notification Form divides severe dengue into three groups: severe plasma leakage, severe bleeding, and severe organ damage. Figure 1 shows that in the severe plasma leakage group, the most prevalent symptom was respiratory failure alone. Some symptoms showed close association, for example, in the group with severe bleeding (Figure 2), there was a higher prevalence of isolated hematemesis and melena, followed by a combination of both. However, symptoms including metrorrhagia and CNS bleeding, showed little association with other symptoms.

**Figure 3:**
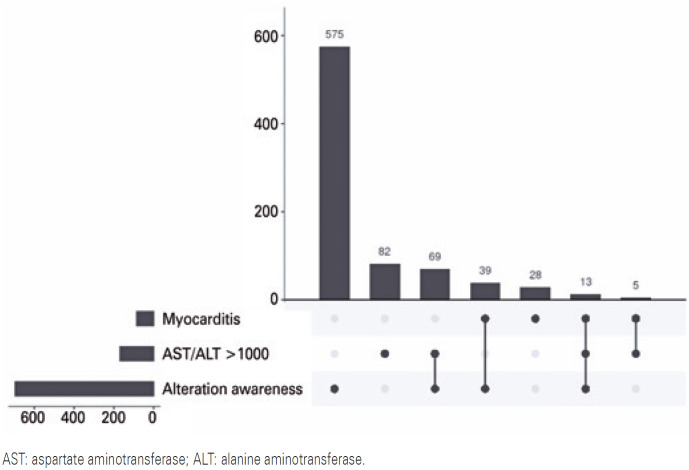
Combination analysis of signs and symptoms of severe organ involvement in pediatric patients with severe dengue in Brazil

In the group with severe organ impairment, the prevalence of alterations in the level of consciousness corroborates the importance of this involvement at the level of the central nervous system (Figure 3). However, there is a low prevalence of hepatic and cardiac involvement, as well as a low joint association of the three symptoms.

**Figure 4:**
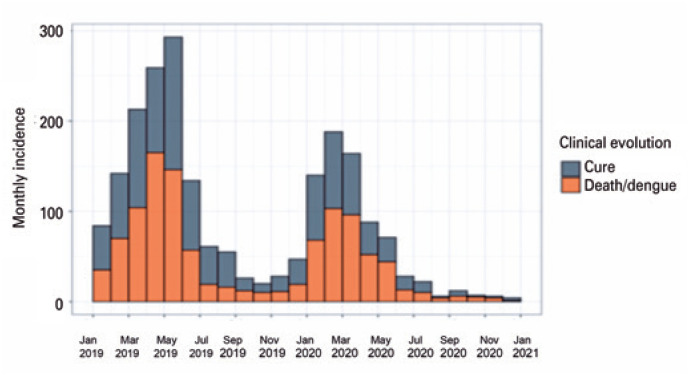
Monthly epidemic curve of confirmed cases of severe dengue among pediatric patients in Brazil

Figure 4 shows the progression of severe dengue cases, categorizing them as fatalities from dengue or cured. This figure demonstrates that fatalities were concentrated in the months of February to May, which confirms the seasonal characteristics of dengue,^(2)^ with a peak in the Brazilian summer and early autumn.

## DISCUSSION

This study of severe dengue in children under the age of 10 aimed to evaluate the seasonal pattern of this tropical disease, its clinical manifestations, progression, and mortality rate.

Based on the data obtained in this study, a high prevalence of dengue was observed in the hot and humid months from January to May as shown in figure 4.^(2)^ This relationship can be explained by the holometabolous character of the mosquito, which requires favorable environmental conditions to undergo metamorphosis through the larval and pupal stages.^(10)^

Although the Midwest region had the highest incidence rate of dengue throughout the nation, from this study, it can be noted that the progression to severe dengue in this location in the pediatric age range was low, 1.6 cases/100 thousand inhabitants. These data can be explained by the fact that the diagnosis of dengue in children is a challenge for professionals, as the clinical manifestations in this population overlap with other conditions in this age group. Therefore, many cases are under- or erroneously reported.^(5)^

It is also possible to conclude that the South and Southeast regions have a high rate of severe dengue, totaling approximately 9.5 cases/100,000 inhabitants and 7.8 cases/100,000 inhabitants, respectively. It is likely that this occurs because this area has a high population concentration with different social conditions, accelerated and uncontrolled growth, without adequate infrastructure for the speed of urban development, and because they are constantly receiving immigrants, which facilitates the proliferation and dissemination of the vector.^(2, 11)^ An article published in *Ciência Geográfica* states that one possible explanation for the prevalence of dengue in the Midwest region is related to urban cleanliness. This analysis was based on data from the Urban Cleaning Sustainability Index (2019), which showed that environmental waste was correctly disposed of in only 18.64% of the region, a lower percentage than in other areas in Brazil.^(11)^

Regarding the symptoms of severe dengue a retrospective cohort study of a reference pediatric hospital in the city of Rio de Janeiro, found that 30.4% of the patients had dyspnea. These data corroborate the results of the present study, in which 29.5% of patients presented with respiratory failure resulting from the accumulation of fluids in the third space. This condition represents an important form of isolated manifestation, as it is a frequent warning sign for volume replacement therapy performed during the critical phase and a complication in the recovery phase. In another study on the clinical-epidemiological profile of severe cases treated at the *Hospital Federal dos Servidores do Estado* of the State of Rio de Janeiro during an epidemic in 2008, plasma leakage was more frequent in children (62.2% of cases) than in other age groups.^(12,13)^ This corroborates the data of this study, as there was a strong relationship between the simultaneous presentation of plasma leakage in this age group, as illustrated in figure 1.

Bleeding manifestations are important in the pediatric age group, as the present study found significant rates of bleeding, mainly gastrointestinal bleeding. Hematemesis alone was the most prevalent presentation of this group of symptoms, accounting for 14.59% of the cases. A study conducted in Sri Lanka with 104 children in 2004 corroborates this, as hematemesis was found in 15% of cases involving the old classification of hemorrhagic fever.^(12)^

When analyzing the manifestations of severe organ impairment, it was observed that the alteration in the level of consciousness, described in the SINAN notification form in isolation, was the most prevalent sign of severe dengue, occurring in approximately 30.9% of cases of pediatric severe dengue. Lethargy is a neurological sign of great importance as a predictor for cases that are candidates for progression to severe dengue, presenting in 47.8% of patients with severe dengue; therefore, early detection of this central nervous system manifestation is vital.^(12)^

Regarding the second most prevalent sign in this group, alterations in liver enzymes (>1000) were observed in 4.41% of patients. In a study carried out with 23 patients aged 2-11 years diagnosed with severe dengue, 39.1% had hepatomegaly and 95.65% had elevated AST (67-118)^(12)^ with a cut-off point of liver damage of AST >40 U/L. The discrepancy between the studies was due to the much higher reference value used in data collection in the present study (AST or ALT values >1000), which followed the severe dengue protocol.^(14)^

According to the SBP, the presence of warning signs of severity should be noted, including severe abdominal pain, persistent vomiting, postural hypotension, drowsiness or irritability, painful hepatomegaly, hemorrhages, abrupt drop in platelets, decreased diuresis, increased hematocrit, respiratory distress, and clinical signs of fluid accumulation (ascites, pleural, and pericardial effusion) to direct the management of dengue.^(7)^ In cases in which such signs were not observed, follow-up was performed on an outpatient basis or in an observation bed. However, at present, follow-ups are performed during hospitalization.^(7)^

Patients with severe dengue, due to the presence of coagulation alterations and plasma extravasation, are categorized red in the Manchester Risk Classification and must be treated immediately, monitored in an emergency bed, with intravenous hydration, laboratory testing, and imaging, according to need.^(7)^

In this study, we observed that the vast majority of cases progressed to hospitalization in corroboration with the SBP protocol for the management of severe dengue.

The progression of dengue is directly related to its diagnosis and adequate treatment according to its classification. The demand for medical care, the limitations and unavailability of diagnostic methods, in addition to the negative impact on the quality of life of patients and their families, are factors that interfere with the difficulty of confirming cases and, consequently, with inadequate management.^(5)^

Diagnosis in the initial phase of the pediatric disease is difficult because the clinical manifestations coincide with other common childhood conditions, such as upper airway infections, enteroviruses, parvoviruses, mononucleosis, acute abdomen, urinary tract infections, pneumonia, rubella, measles, and skin allergies. However, unlike adults, the progression to the severe form occurs suddenly and severe dengue may initially manifest with signs of severity.^(5)^ Regarding outcomes, it was observed that the patients progressed to cure or died from dengue. The number in each group was similar, with 51.2% of deaths and 48.8% of cures observed in 2019 and 2020 in Brazil.

The causes of death are closely linked to complications arising from the symptoms of severe dengue, which involve changes in blood clotting with critical and irreversible systemic repercussions. Thus, the progression of cases to death may be related to severe refractory shock, liver failure, heart failure, encephalitis, and meningitis, among others. Furthermore, failure to recognize alarm signs and inadequate management of complications may result in death due to severe dengue.^(14)^

Among the limitations of this study, there has been an important change in the classification of dengue since 2014, which makes data collection difficult with the new criteria proposed by the World Health Organization (WHO). The review of the diagnosis of dengue by the WHO was proposed due to delays in the results of laboratory criteria previously used and necessary for the classification of cases. After these changes, the new criteria involved clinical signs and symptoms to optimize early treatment, favoring a better prognosis.

Finally, there was a limitation in data collection due to the use of the old classification of dengue hemorrhage, with limited studies being found in this age group that addressed the updated clinical criteria of this pathology.

## CONCLUSION

Severe dengue is more prevalent during the seasonal period with hot and humid characteristics, due to the mechanism involved in the viral and vector cycles. Among the most prevalent symptoms of severe dengue in pediatric patients, respiratory failure alone, gastrointestinal bleeding, and altered level of consciousness were the most frequent. It is important to identify such signs of severity for early intervention, and consequently, for a better prognosis, considering that death is closely related to a delayed diagnosis.

## References

[1] Brasil. Ministério da Saúde (2002). Dengue: aspectos epidemiológicos, diagnóstico e tratament.

[2] Oliveira RM, Oliveira LR (2019). Epidemiologia da Dengue: análise em diversas regiões do Brasil. Rev Cient Esc Sade Exercito.

[3] Zanetti G, Hochhegger B, Marchiori E. (2020). Pulmonary manifestations of dengue. J Bras Pneumol.

[4] Brasil. Ministério da Saúde (2022). Casos graves e óbitos por dengue no Brasil, 2019 a 2022.

[5] Abe HM, Marques SM, Costa PS (2012). Dengue in children: from notification to death. Rev Paul Pediatr.

[6] Brasil. Ministério da Saúde (2009). Guia de vigilância epidemiológica.

[7] Sociedade Brasileira de Pediatria (SBP) (2019). Dengue - Guia prático de atualização.

[8] Borges DX, Nuns EM, Santos ML, Nobre JO (2016). Hemorrhagic dengue: characteristics and importance of early diagnosis. Rev Temas Saúde.

[9] Portilho MM, Lima NV, Caires PS (2021). Alterações hematológicas na dengue grave ­ uma revisão sistemática. Rev Bras Anal Clin.

[10] Brasil. Ministério da Saúde (2001). Dengue - Instruções para Pessoal de Combate ao Vetor - Manual de Normas Técnicas.

[11] Almeida TG, Oliveira ES, Muniz CC (2022). Regionais de saúde e os casos de dengue no mato grosso: a chuva como principal fator para a proliferação do aedes aegypti. Cien Geo.

[12] Pone SM, Hokerberg YH, Oliveira RV, Daumas RP, Pone TM, Pone MV (2016). Clinical and laboratory signs for dengue with severe evolution in hospitalized children. J Pediatr.

[13] Escosteguy CC, Pereira AGL, Medronho RA, Rodrigues CS, Chagas KK (2013). Diferenças, segundo faixa etária, no perfil clínico-epidemiológico dos casos graves de dengue atendidos no Hospital Federal dos Servidores do Estado, Rio de Janeiro-RJ, Brasil, durante a epidemia de 2008. Epidemiol Serv Health.

[14] Brasil. Ministério da Saúde. Secretaria de Vigilância em Saúde. Departamento de Vigilância das Doenças Transmissíveis (2016). Dengue: diagnóstico e manejo clínico: adulto e criança.

